# Estimating undiagnosed dementia in England using capture recapture techniques

**DOI:** 10.1186/s12877-024-05591-0

**Published:** 2025-01-02

**Authors:** Naaheed Mukadam, Louise Marston, Katie Flanagan, Etuini Ma’u, Gary Cheung, Dankmar Böhning

**Affiliations:** 1 University College London, London, UK; 2 University of Auckland, Auckland, New Zealand; 3 University of Southampton, Southampton, UK

**Keywords:** Dementia, Epidemiology, Prevalence

## Abstract

**Background:**

To our knowledge capture-recapture techniques have not been used to estimate dementia prevalence using routinely collected data in England, nor have they been used to estimate changes in undiagnosed dementia over time. In this study we aimed to use routinely collected electronic health records to estimate the number of undiagnosed dementia cases there are in England and how this has changed over time. We also aimed to assess whether proportion of undiagnosed cases differed by age group, ethnicity, socioeconomic deprivation and sex.

**Methods:**

We used routinely collected primary care data linked to hospital episode statistics from 1997 to 2018. We tabulated capture of dementia in each of the two datasets and used the Lincoln-Petersen estimator to estimate numbers of missing dementia diagnoses per year along with the estimated total number of cases and the proportion of cases identified. We calculated age and sex-adjusted prevalence of dementia for each year and used proportion of cases identified to estimate the underlying population prevalence of dementia per year. We conducted beta regression to estimate how sex, age band, deprivation and ethnic group affects the proportion of dementia cases identified, adjusting for year.

**Results:**

Proportion of cases out of the estimated total that were identified, rose from 42.4% in 1997 to 84.4% in 2018. Estimated population prevalence of dementia rose from 1997 to a high of 4.4% in 2018 in those aged ≥ 65. Proportion of dementia cases identified did not vary by sex but a lower proportion of those from the South Asian ethnic group were diagnosed compared to the White population (coeff -0.115, 95% CI -0.218 to -0.011). Compared to those aged 65–74, those aged 75–84 and 85 + had higher proportions of dementia diagnosed (75–84 Coeff 0.259, 95% CI 0.153–0.366; 85 + Coeff 0.185, 95% CI 0.079–0.291). Those living in the two most deprived areas had a higher proportion of dementia diagnosed compared to the least deprived area (IMD quintile 4 vs 1 coeff 0.093, 95% CI 0.014 to 0.173, IMD quintile 5 vs 1 coeff 0.162, 95% CI 0.083 to 0.242).

**Conclusions:**

Proportion of dementia cases identified has increased over time and results indicate that underlying prevalence of dementia may be lower than previously estimated but this needs replication. Greater focus needs to be given to those with dementia onset at younger ages and those from South Asian backgrounds as dementia is relatively under-diagnosed in these groups.

**Supplementary Information:**

The online version contains supplementary material available at 10.1186/s12877-024-05591-0.

## Background

Capture recapture models were originally developed for use in estimating sizes of animal populations. One of the most commonly used, the Lincoln-Petersen model, involves conducting an initial sampling of the animal population and marking those sampled. The animals are then released and the second sampling estimates the size of the total population based on the proportion of the animals that are marked [1]. This principle has been used in estimating undiagnosed/unrecorded diagnoses in humans. By using presence of a particular diagnosis in a dataset, such as routinely collected data or surveys, and looking at the overlap in recording of a diagnosis in different datasets, it is possible to estimate the number of undiagnosed cases. This has been helpful in estimating undiagnosed diabetes [2, 3], and autism [4] among other under-ascertained disorders [5] including mental illness [6]. This kind of estimation can track prevalence of important health conditions without having to conduct repeated, time consuming and expensive epidemiological studies and can help plan health and social services and guide resource allocation. The capture-recapture method has four key assumptions: that the population is closed; that individuals can be matched across different data sources to accurately track capture and recapture; that capture in the different sources of diagnoses is independent of each other; and that the capture probabilities are the same across all individuals in the population [7].

Capture recapture techniques have been used to estimate total dementia prevalence in a 16-year period in Australia [8]. To our knowledge this technique has not been used to estimate dementia prevalence using routinely collected data in England, or other countries, nor has it been used to estimate changes in undiagnosed dementia over time. In this study we aimed to use routinely collected electronic health records to estimate the number of undiagnosed dementia cases in England and how this has changed over time. We also aimed to assess whether the proportion of undiagnosed cases was different by age group, sex, ethnicity and socioeconomic deprivation.

## Method

We pre-registered a protocol for this project before starting analyses (https://osf.io/vkd8q).

### Approvals

We used a fully anonymised dataset from the Clinical Practice Research Datalink (CPRD) which has National Research Ethics Service Committee (NRES) approval for purely observational research using primary care data and established data linkages.

The study was approved by the Independent Scientific Advisory Committee (ISAC) of the Medicines and Healthcare Products Regulatory Agency (MHRA) (protocol 19_235).

### Datasets

This study used the CALIBER © resource (https://www.ucl.ac.uk/health-informatics/caliber and https://www.caliberresearch.org/) which provides data management of CPRD electronic primary care records linked with hospital and mortality records. All patients have a unique patient identifier which allows linkage across all datasets. We had planned to use all three data sources for estimates, but the mortality data had very few cases of dementia and did not have any cases independent of the other two sources. This was despite including any cases of dementia, even if not listed as primary cause of death. Therefore, we limited analyses of dementia diagnosis to two datasets only – primary care records and hospital data (Hospital Episode Statistics – HES) but included mortality data to determine date of death. Data are collected from routinely recorded electronic health records from primary care practices who consent to data collection and is linked to hospital data (including details of hospital admissions, outpatient appointments and presentations to Accident and Emergency). Records from primary care include codes related to diagnoses and medications, while records from hospital includes ICD-10 codes for diseases that patients have or are diagnosed with during their presentation. Codes used to identify dementia in both sources are available online (https://osf.io/tvau4/).

### Sample

Most people living in England are registered with a General Practitioner (GP) and people in primary care databases are generally representative of the general population, including in ethnicity [9]. As linked electronic hospital records are country-specific and are only provided for GP practices in England which have consented to participate in the CPRD patient-level linkage scheme [10], we restricted analyses to practices from England consenting to the patient-level linkage scheme (around 88% of practices overall [11]). We focused our analyses on dementia diagnosed after reaching age 65, as dementia is unusual below this age and aetiology of younger onset dementia is usually substantially different [12]. The start date for each participant was the latest of their 65th birthday, 1st January 1997, date of registration with the GP practice or when the GP practice data met data quality standards. The end date for each participant was the earliest of: date of last data collection for the practice; patient transfer out of the practice; death; or the end of study (31st December 2018).

### Variables

#### Dementia

We considered all-cause dementia as our main outcome, defined as the first record of any diagnostic code or anti-dementia medication recorded in either HES or CPRD. Cases continued to be counted after identification for as long as the person was alive, ie counting prevalent dementia.

#### Age and sex

Date of birth and sex as defined in primary care records were used to stratify analyses based on age band and sex.

#### Deprivation

We used Index of Multiple Deprivation (IMD) as a measure of socioeconomic deprivation. This is a composite measure available from primary care data, based on postcode and derived from a number of indicators covering domains of material deprivation: income, employment, education and skills, health, housing, crime, access to services, and living environment [13].

#### Ethnicity

Ethnicity is recorded in CPRD, as well as HES. Patients can self-identify their ethnic group and codes are entered into records by healthcare professionals. We chose to classify by ethnicity instead of race as the NHS does not collect data on “race”. Furthermore, while race primarily defines biological characteristics, ethnicity is a broader construct encompassing common cultural traits. We used ethnicity as defined in either source of data at any time. When ethnicity is recorded in more than one database, the agreement between ethnicities is high [14]. Where conflict existed between CPRD and HES, priority was given to the CPRD classification because CPRD ethnicity reflects population percentages [14] whereas HES ethnicity is less accurate [15]. Ethnicity categories are those used in the UK Census, which includes 17 ethnic groups: White British, White Irish, White Gypsy, White Other, Asian Bangladeshi, Asian Indian, Asian Pakistani, Asian Chinese, Asian Other, Black African, Black Caribbean, Black other, Mixed White and Black Caribbean, Mixed White and Black African, Mixed White and Asian, Any other mixed background, Other (Arab), Other (any other ethnic group). Both CPRD and HES use these categories.

We combined ethnicities into White (all white groups); South Asian (Bangladeshi, Indian, Pakistani) and Black (Black Caribbean, Black African and Black British). All other ethnic groups including all mixed ethnic groups were combined into a separate group and included in analyses but not reported as we focused on the three main ethnic groups and the fourth group contained large within-group heterogeneity. We stratified analyses by ethnic group where possible.

#### Analysis

All analyses were conducted in Stata Version 17.0 and the software R (R Version 4.3.1).

We recorded the earliest recorded dementia diagnosis or indication of prescription for anti-dementia medication for each person in each dataset (primary care, hospital records). For each year, we calculated how many people had been diagnosed with dementia and in which dataset their diagnosis occurred. Cases were counted as belonging to a calendar year if diagnosis was prior to 15th April of that year (from any source), as that date is after Quality Outcomes Framework data (key data from primary care, including dementia) from GPs is due. Cases were only counted if each participant also had valid records starting before and ending after 15th April of the index year. We then tabulated the different combinations of diagnoses across the two datasets. We did this for the whole sample for each year and repeated the process by sex and by 10 year age bands (65–74, 75–84, 85 +). We did the same by ethnic group and IMD quintile.

Table 1 shows the combinations of case capture that are in the dataset, with the aim being to calculate the missing value.

**Table 1:** identified and missing cases used in Lincoln-Petersen approach

Count	Primary Care	Hospital Records
A	1	1
B	1	0
C	0	1
M (Missing value estimated by modelling)	0	0

A = the count of cases identified by Primary Care and Hospital Records, B = cases only identified by Primary care but not by Hospital Records, and C = cases only identified by Hospital Records but not Primary Care; M are those unknown cases missed by both

As only two lists or sources, namely primary care and hospital records, were available, the only available estimator is based on the Lincoln-Petersen approach [16]. This works as follows. Under independence of sources, which is a crucial assumption, the odds ratio of case capture based on Table 1 is:

$$\mathrm{OR}\;=\;(\mathrm A\;\mathrm x\;\mathrm M)/(\mathrm B\;\mathrm x\;\mathrm C).$$

The meaning of A, B, C, and M have been explained in Table 1. For example, A stands for the frequency count arising from Primary Care and Hospital Records.

Under the assumption of independence, the OR is 1, so that the above equation has the solution:

$$\mathrm M\;=\;(\mathrm B\;\mathrm x\;\mathrm C)/\mathrm A$$

for the missing cell frequency. This is the Lincoln-Petersen estimator [17, 18]. Chapman suggested an improved estimator by replacing A by (A + 1). The associated Chapman estimator of the population size of dementia is then N = A + B + C + (B x C)/(A + 1) [19]. This formula is used to determine observed and unobserved dementia cases for each year under consideration and all combinations of demographic strata.

We reported numbers of missing dementia diagnoses per year along with the estimated total number of cases and the proportion of cases identified. Proportion of cases identified were calculated as (identified cases)/(identified + missing cases). We also reported these figures stratified by sex, age band, IMD and ethnic group with 95% confidence intervals. We devoted considerable effort in constructing confidence intervals of which details are given in the Appendix. We conducted beta regression to estimate how sex, age band, IMD and ethnic group affects the proportion of dementia cases identified, adjusting for year, using the Stata command betareg. Positive coefficients from this regression indicate higher proportion of identified cases while negative coefficients indicate lower proportion of cases identified relative to the reference category. We calculated overall crude prevalence (and 95% CIs) for the whole sample per year from 1997 to 2018. We then used the English population structure for 2018 [20] to produce age and sex standardised point prevalence estimates for each year.

## Results

**Fig. 1:**
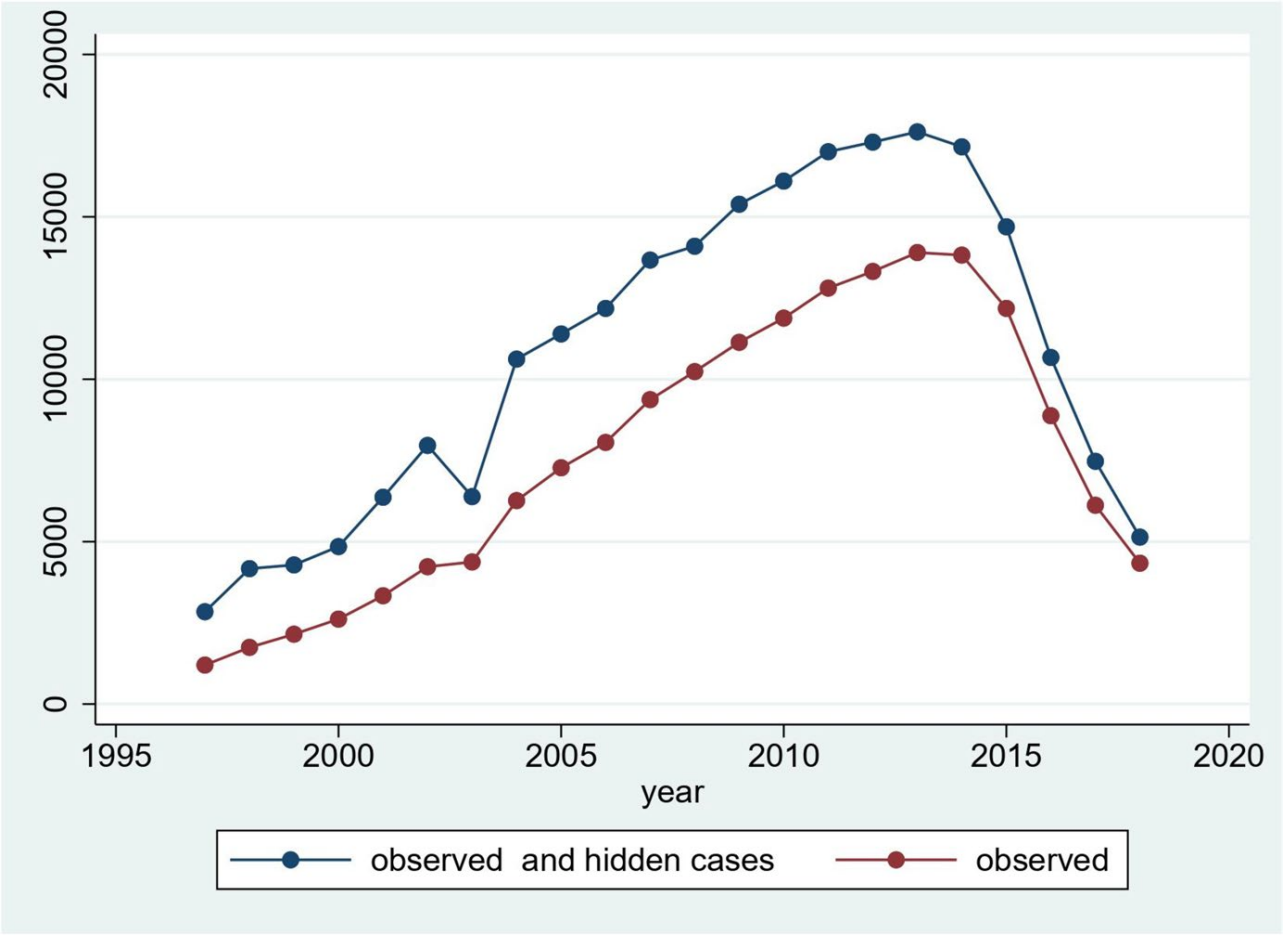
Total and observed cases over time

**Fig. 2:**
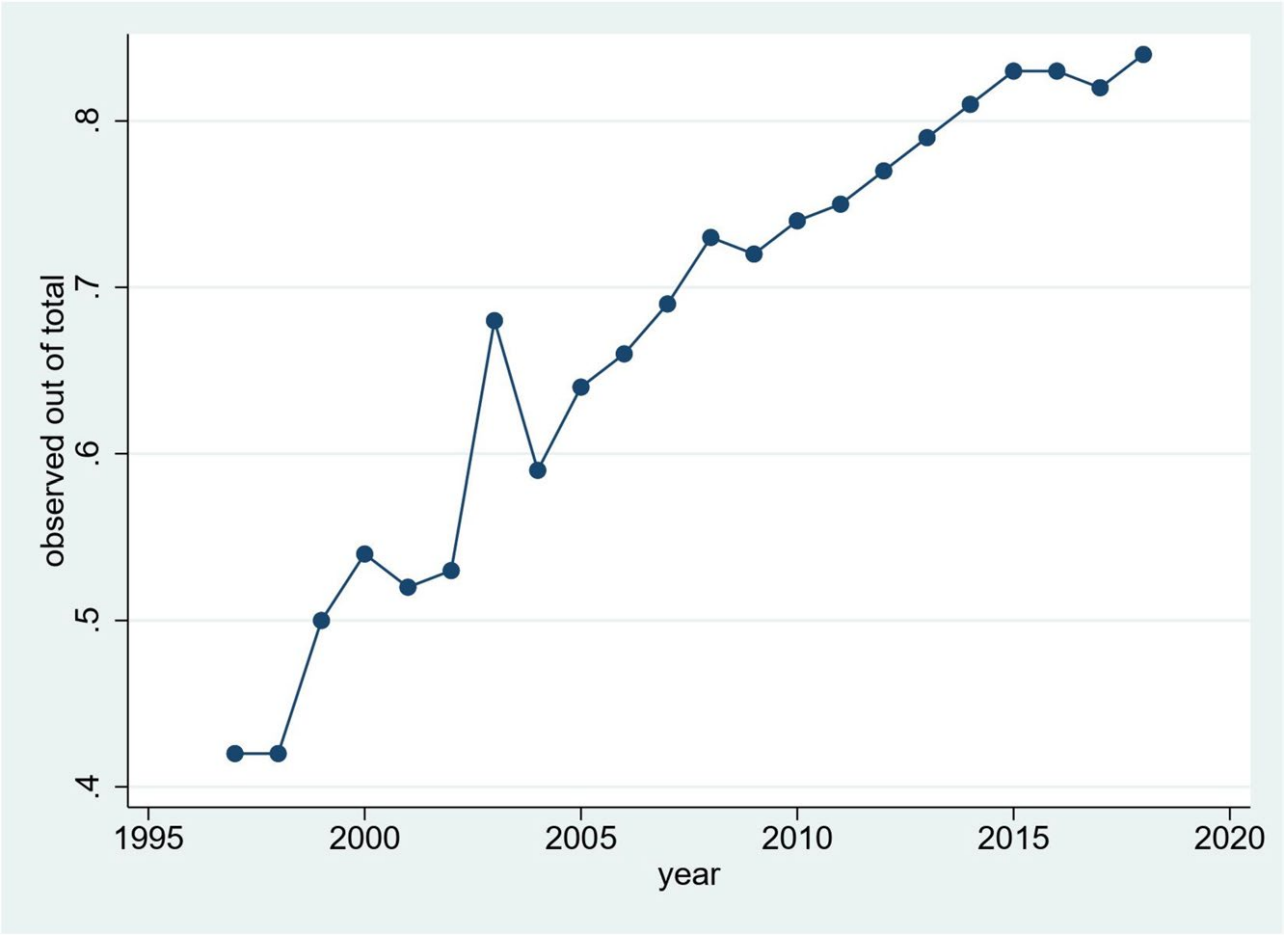
Proportion of observed cases over time

The number of English GP practices contributing data rose from 146 in 1997 to 360 in 2008 then fell to 89 in 2018. Total dementia cases and observed cases rose steadily from 1200 in 1997 to 13,901 in 2013 then declined to 4337 in 2018. The proportion of cases out of the estimated total that were identified, rose from 42.4% in 1997 to 84.4% in 2018. These trends are shown in Figs. 1 and 2 respectively with crude numbers in the Appendix. Cases identified in both datasets was generally less than 20% of total observed cases. Raw data on case tabulations is available online (https://osf.io/tvau4/files/osfstorage). Crude and adjusted prevalence increased from 1997 to 2018 (see Appendix). In 2018, the age and sex-adjusted estimated prevalence, for those aged 65 + , taking into account uncaptured cases, was 4.4%.

**Fig. 3:**
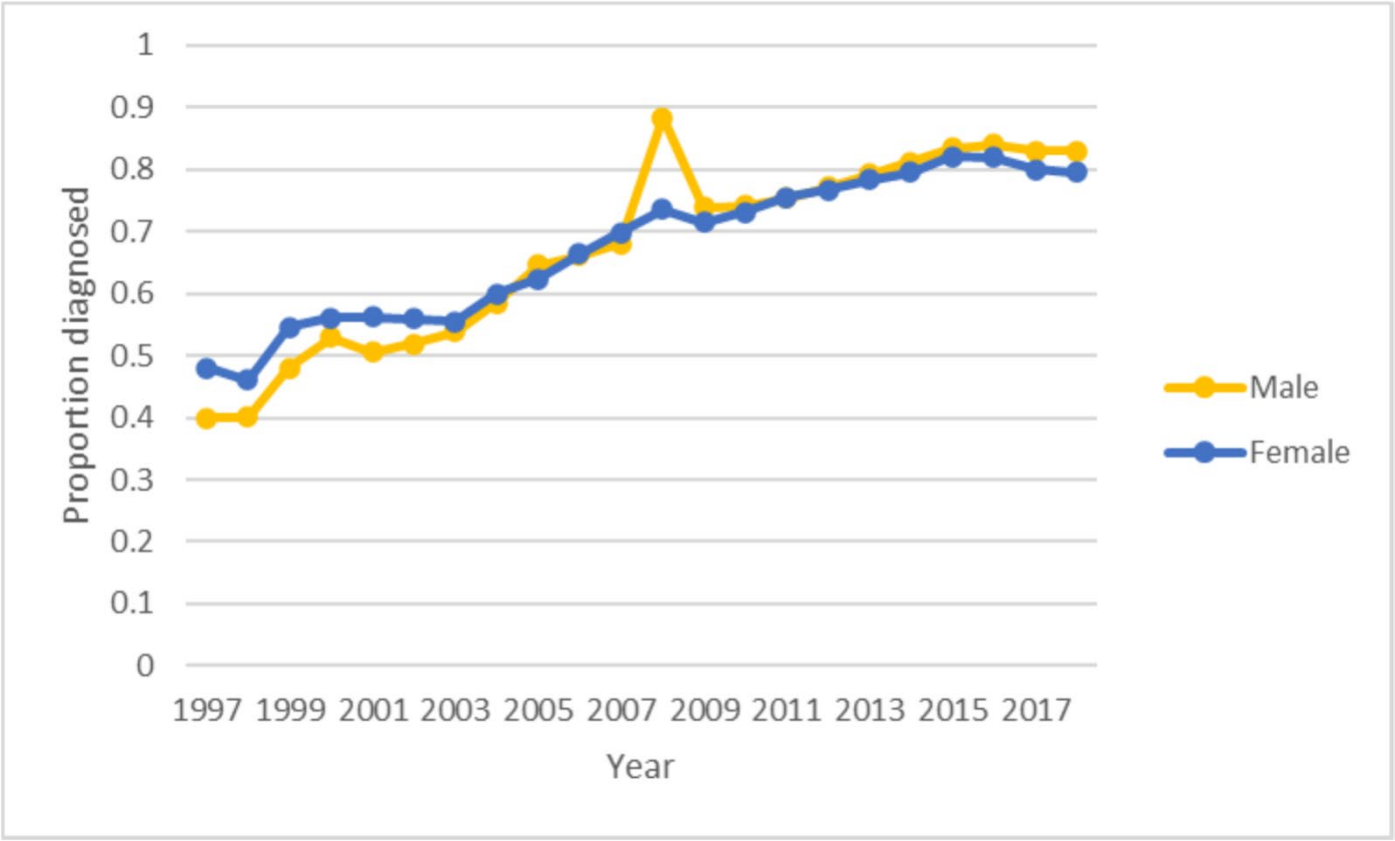
Proportion of cases diagnosed by sex

**Fig. 4:**
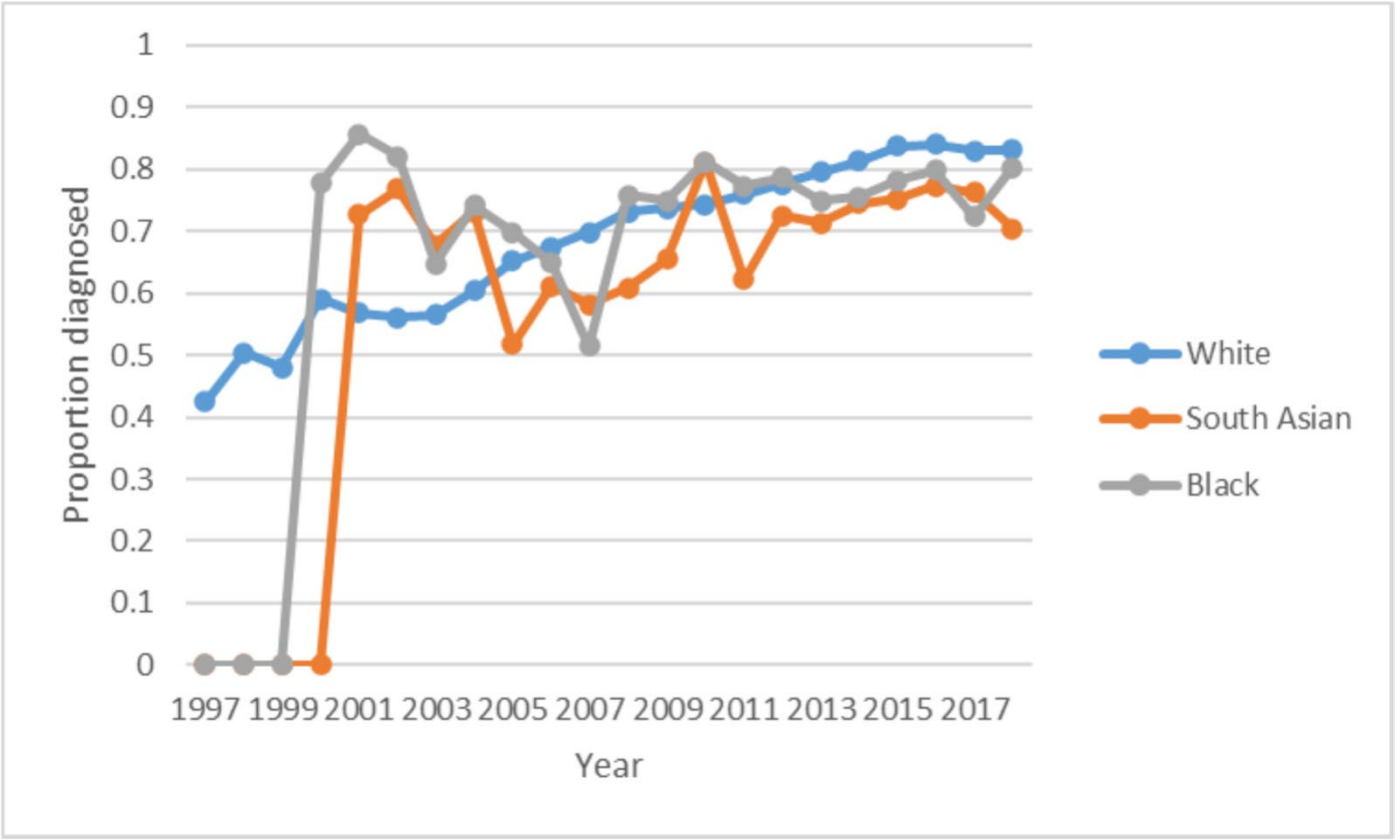
Proportion of cases diagnosed by ethnicity

Proportion diagnosed by sex increased over time for both sexes with female proportion slightly lower than male by 2018 (Fig. 3). Regression analysis showed no difference in proportion diagnosed by sex (Coeff male vs female 0.001, 95% CI -0.122 to 0.124). There was no data for the first few years for the South Asian and Black groups. Proportion of cases diagnosed increased steadily from 1997 to 2018 for people from White ethnic backgrounds. Proportion of cases diagnosed for Black and South Asian ethnic groups was high initially when sufficient data became available in 1999/2000 but then rates were roughly stable with some variation around the mean. Proportion diagnosed was generally lower for minority ethnic groups, particularly from 2013 onwards (Fig. 4). South Asians had a lower proportion of cases diagnosed (coefficient -0.115, 95% CI -0.218 to -0.011) whereas there were no significant differences in the Black versus White group (coefficient -0.037, 95% CI -0.141 to 0.067).

**Fig. 5:**
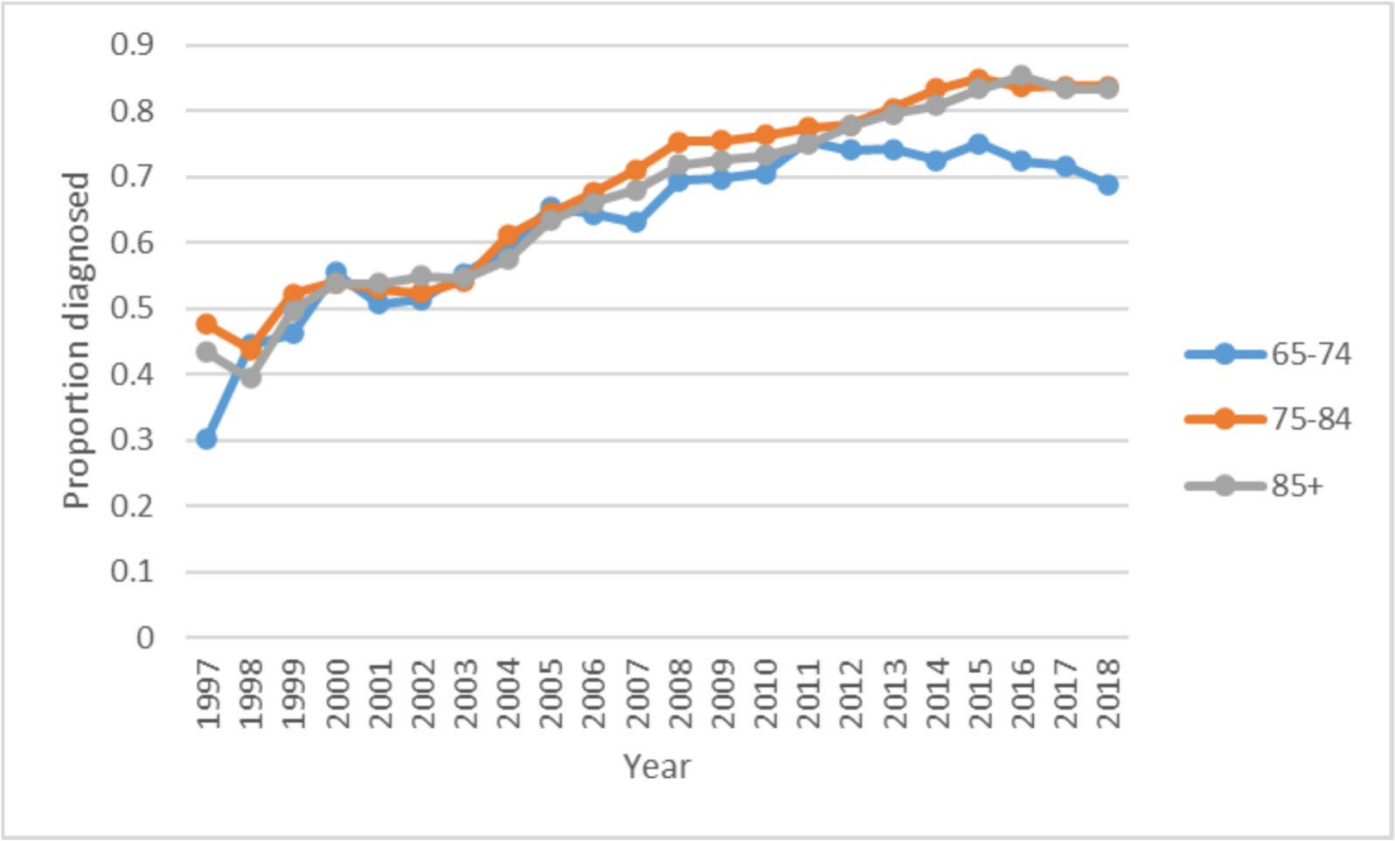
Proportion of cases diagnosed by age group

Proportion diagnosed increased for all age groups over time initially and continued to rise for the two older age groups (75–84 and 85 +), but levelled off in 2010 and then decreased for the youngest age group (65–74 years) from 2015 (see Fig. 5). Compared to those aged 65–74, those aged 75–84 and 85 + had higher proportions of dementia diagnosed, (75–84 Coeff 0.259, 95% CI 0.153–0.366; 85 + Coeff 0.185, 95% CI 0.079–0.291).

**Fig. 6:**
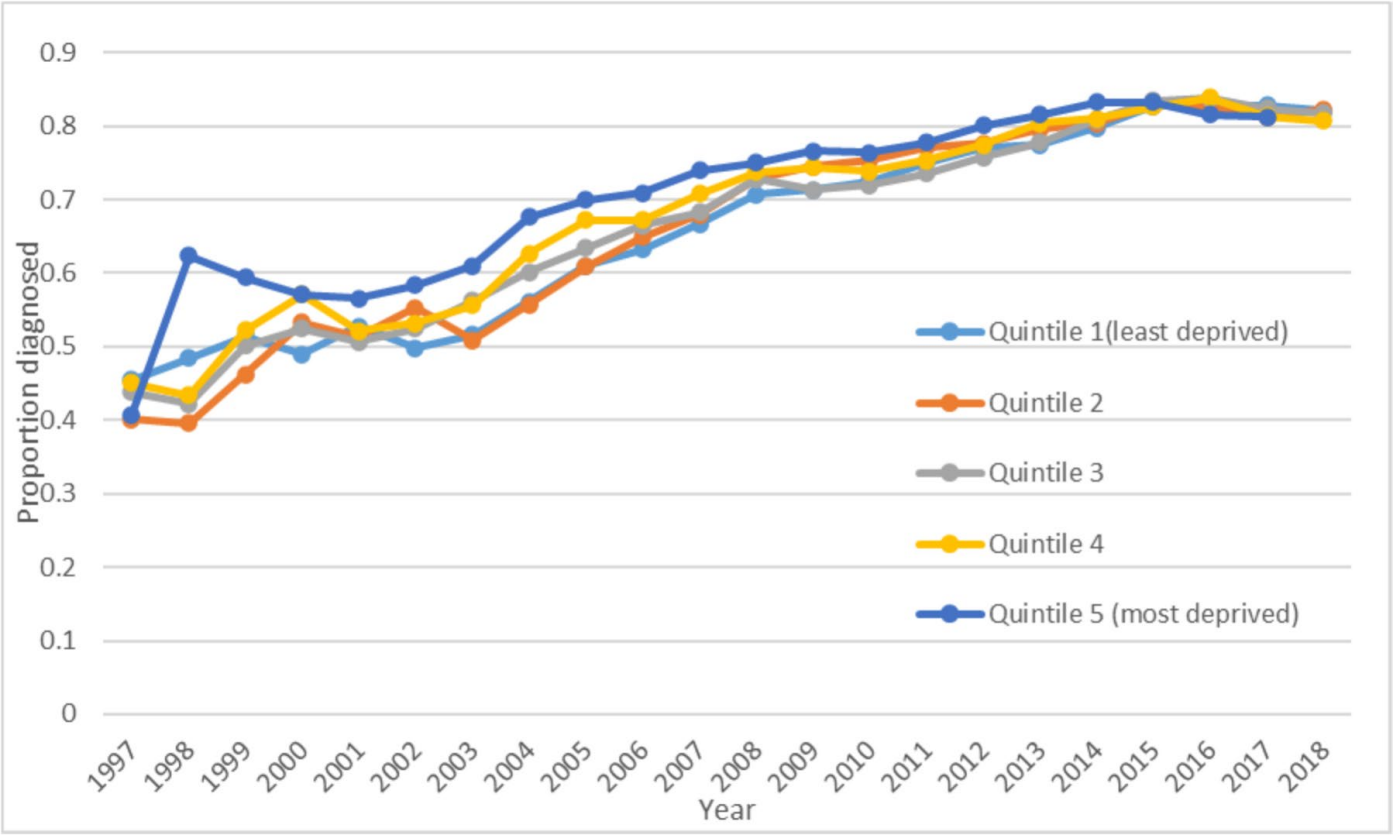
Proportion of cases diagnosed by IMD quintile

Proportion diagnosed increased over time for all IMD quintiles (Fig. 6). When results were analysed by IMD, only those living in the two most deprived areas had a higher proportion of dementia diagnosed compared to the least deprived areas (IMD quintile 4 vs 1 coeff 0.093, 95% CI 0.014 to 0.173, IMD quintile 5 vs 1 coeff 0.162, 95% CI 0.083 to 0.242).

## Discussion

In this study we estimate for the first time, the proportion of dementia diagnosed over time and how this varies by sociodemographic characteristics. We find the proportion of dementia diagnosed has risen continuously since 1997 and is now in excess of 80%. The initial rise could be due to increasing awareness of dementia and approval of donepezil and other cholinesterase inhibitors for management of mild to moderate dementia from 1997 onwards [21]. Later increases could be due to policy initiatives to increase the diagnosis of dementia, such as incentivising recording of dementia diagnoses in primary care by making a dementia register in practices part of the Quality and Outcomes Framework in 2005–6 [22], the 2009 National Dementia Strategy [23] and 2012 Prime Minister’s Challenge on dementia [24]. The national target for the proportion of dementia diagnosed is 66.7% and is calculated monthly by the NHS by dividing the recorded number of cases from primary care by the expected number based on population based estimates from the 2011 Cognitive Function and Ageing Study (CFAS) [25]. This figure has previously been achieved but has fallen post-pandemic to 64.8% as of March 2024 [26]. Our results are not completely comparable as we use both primary care and hospital data to calculate prevalence. Reporting of hospital data is not as contemporaneous as primary care, so it is not clear if it would be possible to conduct this analysis every month as is currently done.

We were able to assess whether sex, age, deprivation and ethnicity affect the proportion of dementia cases diagnosed. It was concerning that the proportion of younger people (aged 65–74) with dementia who are diagnosed is lower than those in older age bands. This may be because younger people with dementia are less likely to be in contact with health services due to better health overall but may also be due to healthcare professionals being less likely to think of dementia as a possibility in relatively young older adults. Those of South Asian ethnicity have a lower proportion of diagnoses compared to the White population. This may be as people from South Asian ethnicity may be more reluctant to seek help for dementia due to stigma or perceived discrimination from the healthcare system [27]. People from the most socioeconomically deprived areas have a higher proportion of diagnoses compared to those from least deprived areas. This may be due to higher rates of multimorbidity in those from more deprived areas [28], meaning they are more likely to be in contact with primary care services and therefore may be more likely to have their cognitive impairment noticed and recorded by healthcare professionals. People with higher levels of deprivation are also more likely to be hospitalised and to have longer hospital admissions [29], again increasing the chances of their dementia being identified and recorded.

The main limitation of this work is that ultimately estimates are dependent on dementia diagnoses being captured in healthcare records. Prevalence estimates are very low, particularly in the earlier years of data collection with estimates less than half that of CFAS population based surveys for the same time period [25]. This is likely a reflection of the relative under-capture of dementia in records overall. Although capture-recapture should be able to compensate for this by using multiple sources of data, it is possible that if overall capture in both datasets is low, that this leads to underestimates. In addition, the capture-recapture methods assume a closed population, that capture in the different sources of diagnoses is independent of each other; and that the capture probabilities are the same across all individuals in the population [7]. These assumptions may not be true. CPRD is a dynamic cohort, with people entering and leaving the cohort at different times. While we have relative stability within each year where we estimate prevalence, overall the population is not closed. To account for this we have stratified the analysis by year which seems a reasonable approach to achieve a closed population as the condition is usually not of short duration. In England, when people are discharged from hospital, correspondence will be sent from the hospital to the patient’s primary care provider. This correspondence may list a new dementia diagnosis and these could then be captured in the primary care records. Likewise, diagnoses from primary care would be on patient records and would then also be entered into hospital data. In this way, the two sources of dementia capture may not be completely independent and there may also be differences in capture probabilities for patients depending on their other health conditions and available healthcare facilities. This may have led to underestimates of missing cases and low prevalence estimates, with previous work highlighting that life sciences almost always deal with positive dependence between data sources [16]. 

As recording of dementia has been incentivised and encouraged in primary care in England since 2006 [22], our recording of dementia diagnoses has improved. Diagnostic practices have also changed over this time period, meaning diagnoses may be more accurate now than previously. It is possible that dementia prevalence has declined since previous population-based estimates. Other studies using survey data have suggested dementia incidence may be declining [30]. Or it may be that this method of estimating prevalence is not accurate and better quality dementia diagnosis capture and independent data sources are required. Another limitation is that practices contributing data to the analysis declined significantly over the survey period. They may have stopped contributing due to changes in computer systems, not meeting data quality standards or removal of consent to data linkage and it is not possible to determine whether these practices differ from the practices that continued contributing data. Although this would have led to fewer cases being identified overall, it should not affect prevalence of dementia unless the practices that dropped out differed in their populations from the practices that continued to contribute data. Our data was from England but methods used can be applied to other healthcare systems which use electronic health records and have the possibility of dementia being captured in multiple data sources.

## Conclusions

Overall, our results suggest that more focus is needed on ensuring parity with regards to dementia diagnosis, particularly in South Asian people and among those who have dementia at relatively younger ages. Further work is also required in validating our findings and estimating more updated prevalence and incidence figures.

## Supplementary information

Supplementary material 1.

## Data Availability

All data are available at OSF at the link listed in the manuscript.

## References

[1] Seber GAF. The estimation of animal abundance and related parameters: Blackburn press Caldwell, New Jersey; 1982.

[2] Gill GV, Ismail AA, Beeching NJ, Macfarlane SB, Bellis MA. Hidden diabetes in the UK: use of capture-recapture methods to estimate total prevalence of diabetes mellitus in an urban population. J R Soc Med. 2003;96(7):328–32. 12835444 10.1258/jrsm.96.7.328 PMC539535

[3] Cameron CM, Coppell KJ, Fletcher DJ, Sharples KJ. Capture-recapture using multiple data sources: estimating the prevalence of diabetes. Aust N Z J Public Health. 2012;36(3):223–8. 22672027 10.1111/j.1753-6405.2012.00868.x

[4] Harrison MJ, O’Hare AE, Campbell H, Adamson A, McNeillage J. Prevalence of autistic spectrum disorders in Lothian, Scotland: an estimate using the “capture-recapture” technique. Arch Dis Child. 2006;91(1):16–9. 15886261 10.1136/adc.2004.049601 PMC2083098

[5] Chao A, Tsay P, Lin SH, Shau WY, Chao DY. The applications of capture-recapture models to epidemiological data. Stat Med. 2001;20(20):3123–57. 11590637 10.1002/sim.996

[6] Fisher N, Turner SW, Pugh R, Taylor C. Estimating numbers of homeless and homeless mentally ill people in north east Westminster by using capture-recapture analysis. BMJ. 1994;308(6920):27–30. 8298348 10.1136/bmj.308.6920.27 PMC2539171

[7] Yip P, Bruno G, Tajima N, Seber G, Buckland S, Cormack R, et al. Capture-recapture and multiple-record systems estimation I: history and theoretical development. Am J Epidemiol. 1995;142(10):1047–58. 7485050

[8] Waller M, Mishra GD, Dobson AJ. Estimating the prevalence of dementia using multiple linked administrative health records and capture-recapture methodology. Emerg Themes Epidemiol. 2017;14:3. 28261312 10.1186/s12982-017-0057-3 PMC5327574

[9] Walley T, Mantgani A. The UK general practice research database. The Lancet. 1997;350(9084):1097–9. 10213569 10.1016/S0140-6736(97)04248-7

[10] Datalink CPR. Small area level data based on patient postcode. 2020. p. https://cprd.com/sites/default/files/Documentation_SmallAreaData_Patient_set21_v3.0.pdf. Accessed May 2024.

[11] Padmanabhan S, Carty L, Cameron E, Ghosh RE, Williams R, Strongman H. Approach to record linkage of primary care data from Clinical Practice Research Datalink to other health-related patient data: overview and implications. Eur J Epidemiol. 2019;34(1):91–9. 30219957 10.1007/s10654-018-0442-4 PMC6325980

[12] van der Flier WM, Scheltens P. Epidemiology and risk factors of dementia. J Neurol Neurosurg Psychiatry. 2005;76(suppl 5):v2–7. 16291918 10.1136/jnnp.2005.082867 PMC1765715

[13] McLennan D, Noble S, Noble M, Plunkett E, Wright G, Gutacker N. The English indices of deprivation 2019: Technical report. 2019.

[14] Mathur R, Bhaskaran K, Chaturvedi N, Leon DA, vanStaa T, Grundy E, Smeeth L. Completeness and usability of ethnicity data in UK-based primary care and hospital databases. J Public Health. 2013;36(4):684–92. 10.1093/pubmed/fdt116 PMC4245896 24323951

[15] Saunders CL, Abel GA, El Turabi A, Ahmed F, Lyratzopoulos G. Accuracy of routinely recorded ethnic group information compared with self-reported ethnicity: evidence from the English Cancer Patient Experience survey. BMJ Open. 2013;3(6): e002882. 23811171 10.1136/bmjopen-2013-002882 PMC3696860

[16] Brittain S, Böhning D. Estimators in capture–recapture studies with two sources. AStA Advances in Statistical Analysis. 2009;93(1):23–47.

[17] Petersen CGJ. The yearly immigration of young plaice in the Limfjord from the German sea. Rept Danish Biol Sta. 1896;6:1–48.

[18] Lincoln FC. Calculating waterfowl abundance on the basis of banding returns. Washington, USA: US Department of Agriculture; 1930.

[19] Chapman DG. Some properties of the hypergeometric distribution with applications to zoological censuses. Univ Calif Stat. 1951;1:60–131.

[20] Office for National Statistics. Population estimates for the UK, England and Wales, Scotland and Northern Ireland: mid-2018. 2019.

[21] Rogers SL, Friedhoff LT. The efficacy and safety of donepezil in patients with Alzheimer’s disease: results of a US Multicentre, Randomized, Double-Blind, Placebo-Controlled Trial, The Donepezil Study Group. Dementia. 1996;7(6):293–303. 8915035 10.1159/000106895

[22] NHS Digital. Quality and Outcomes Framework. 2023.

[23] Department of Health. Living well with dementia: A national dementia strategy: Department of Health; 2009.

[24] Department of Health. Prime Minister's challenge on dementia 2020. 2015.

[25] Matthews FE, Arthur A, Barnes LE, Bond J, Jagger C, Robinson L, et al. A two-decade comparison of prevalence of dementia in individuals aged 65 years and older from three geographical areas of England: results of the Cognitive Function and Ageing Study I and II. The Lancet. 2013;382(9902):1405–12. 23871492 10.1016/S0140-6736(13)61570-6 PMC3906607

[26] NHS England. Primary care dementia data 2017 [Available from: https://digital.nhs.uk/data-and-information/publications/statistical/primary-care-dementia-data. Accessed May 2024.

[27] Mukadam N, Cooper C, Basit B, Livingston G. Why do ethnic elders present later to UK dementia services? A qualitative study International Psychogeriatrics. 2011;23(7):1070–7. 21349212 10.1017/S1041610211000214

[28] Pathirana TI, Jackson CA. Socioeconomic status and multimorbidity: a systematic review and meta-analysis. Aust N Z J Public Health. 2018;42(2):186–94. 29442409 10.1111/1753-6405.12762

[29] Luben R, Hayat S, Khawaja A, Wareham N, Pharoah PP, Khaw K-T. Residential area deprivation and risk of subsequent hospital admission in a British population: the EPIC-Norfolk cohort. BMJ Open. 2019;9(12): e031251. 31848162 10.1136/bmjopen-2019-031251 PMC6937051

[30] Ahmadi-Abhari S, Kivimäki M. Do age-standardised dementia incidence rates really increase in England and Wales? Lancet Public Health. 2024;9(3):e152–3. 38429014 10.1016/S2468-2667(24)00019-7

