# Supplementary material for: Estimating undiagnosed dementia in England using capture recapture techniques
Source: BMC Geriatr. 2025 Jan 2;25:1. doi: 10.1186/s12877-024-05591-0 (PMC11694474; doi:10.1186/s12877-024-05591-0)
Supplement: Supplementary file 1 — Supplementary material 1. [file 12877_2024_5591_MOESM1_ESM.zip › 12877_2024_5591_MOESM1_ESM.docx]

# Estimating undiagnosed dementia in England using capture recapture techniques: supplementary material

**Uncertainty Assessment of the Chapman Estimator**

Our approach in constructing valid 95% confidence intervals is as follows.

After filling in the missing value for M in Table 1 we can draw samples from the Tables of size N = A + B + C + (B x C)/(A+1).

Each sample is of the form A*, B*, C*, M*.

We drop M* and calculate the associated Chapman estimator N* = A* + B* + C* + (B* x C*)/(A*+1). This process is repeated R times with R being large such as R=10,000. This gives us a sample of size R with replications of N*.

We determine from this sample a 95% confidence interval by taking the 2.5^th^ percentile as lower limit and the 97.5^th^ percentile as upper limit. We provide the associated R code as web-supplement.

**Table S1: Crude results – proportion of dementia cases diagnosed**

| **Year** | **Observed** | **Estimated Total** | **Estimated Total LCL** | **Estimated Total UCL** | **LCL Proportion** | **Proportion observed** | **UCL proportion** |
| --- | --- | --- | --- | --- | --- | --- | --- |
| 1997 | 1200 | 2842 | 2164 | 3606 | 0.333 | 0.422 | 0.554 |
| 1998 | 1744 | 4169 | 3733 | 4705 | 0.371 | 0.418 | 0.467 |
| 1999 | 2150 | 4284 | 3977 | 4634 | 0.464 | 0.502 | 0.541 |
| 2000 | 2615 | 4848 | 4566 | 5160 | 0.507 | 0.539 | 0.573 |
| 2001 | 3335 | 6367 | 6023 | 6750 | 0.494 | 0.524 | 0.554 |
| 2002 | 4228 | 7962 | 7593 | 8377 | 0.505 | 0.531 | 0.557 |
| 2003 | 4375 | 6387 | 6166 | 6615 | 0.661 | 0.685 | 0.710 |
| 2004 | 6263 | 10621 | 10253 | 11000 | 0.569 | 0.590 | 0.611 |
| 2005 | 7275 | 11395 | 11071 | 11738 | 0.620 | 0.638 | 0.657 |
| 2006 | 8055 | 12180 | 11868 | 12503 | 0.644 | 0.661 | 0.679 |
| 2007 | 9373 | 13670 | 13363 | 13974 | 0.671 | 0.686 | 0.701 |
| 2008 | 10235 | 14095 | 13822 | 14367 | 0.712 | 0.726 | 0.741 |
| 2009 | 11137 | 15388 | 15116 | 15669 | 0.711 | 0.724 | 0.737 |
| 2010 | 11882 | 16101 | 15831 | 16382 | 0.725 | 0.738 | 0.751 |
| 2011 | 12807 | 17005 | 16732 | 17271 | 0.742 | 0.753 | 0.765 |
| 2012 | 13319 | 17300 | 17053 | 17553 | 0.759 | 0.770 | 0.781 |
| 2013 | 13901 | 17618 | 17381 | 17862 | 0.778 | 0.789 | 0.800 |
| 2014 | 13821 | 17156 | 16941 | 17380 | 0.795 | 0.806 | 0.816 |
| 2015 | 12182 | 14694 | 14503 | 14887 | 0.818 | 0.829 | 0.840 |
| 2016 | 8877 | 10671 | 10511 | 10839 | 0.819 | 0.832 | 0.845 |
| 2017 | 6121 | 7475 | 7335 | 7620 | 0.803 | 0.819 | 0.834 |
| 2018 | 4337 | 5140 | 5042 | 5245 | 0.827 | 0.844 | 0.860 |

**LCL – lower confidence limit, UCL - upper confidence limit**

**Table S2: Age and sex-adjusted prevalence**

| **Year** | **Crude prevalence (%)** | **Age and sex adjusted prevalence (%)** | **Age and sex adjusted prevalence LCL** | **Age and sex adjusted prevalence UCL** | **Proportion diagnosed** | **Estimated true prevalence*** | **Estimated true prevalence LCL** | **Estimated true prevalence UCL** |
| --- | --- | --- | --- | --- | --- | --- | --- | --- |
| 1997 | 0.6 | 0.6 | 0.5 | 0.6 | 0.4 | 1.4 | 1.3 | 1.4 |
| 1998 | 0.8 | 0.7 | 0.7 | 0.8 | 0.4 | 1.8 | 1.7 | 1.8 |
| 1999 | 0.8 | 0.8 | 0.7 | 0.8 | 0.5 | 1.5 | 1.4 | 1.6 |
| 2000 | 0.7 | 0.7 | 0.7 | 0.7 | 0.5 | 1.3 | 1.3 | 1.4 |
| 2001 | 0.9 | 0.8 | 0.8 | 0.8 | 0.5 | 1.5 | 1.5 | 1.6 |
| 2002 | 1.0 | 0.9 | 0.9 | 0.9 | 0.5 | 1.7 | 1.7 | 1.8 |
| 2003 | 1.1 | 1.1 | 1.0 | 1.1 | 0.7 | 1.6 | 1.5 | 1.6 |
| 2004 | 1.3 | 1.3 | 1.2 | 1.3 | 0.6 | 2.1 | 2.1 | 2.2 |
| 2005 | 1.5 | 1.4 | 1.4 | 1.4 | 0.6 | 2.2 | 2.1 | 2.2 |
| 2006 | 1.6 | 1.5 | 1.5 | 1.5 | 0.7 | 2.3 | 2.2 | 2.3 |
| 2007 | 1.8 | 1.7 | 1.7 | 1.7 | 0.7 | 2.5 | 2.4 | 2.5 |
| 2008 | 2.0 | 1.8 | 1.8 | 1.9 | 0.7 | 2.5 | 2.5 | 2.6 |
| 2009 | 2.1 | 2.0 | 1.9 | 2.0 | 0.7 | 2.7 | 2.7 | 2.8 |
| 2010 | 2.3 | 2.1 | 2.1 | 2.2 | 0.7 | 2.9 | 2.9 | 3.0 |
| 2011 | 2.5 | 2.4 | 2.3 | 2.4 | 0.8 | 3.1 | 3.1 | 3.2 |
| 2012 | 2.6 | 2.5 | 2.5 | 2.6 | 0.8 | 3.3 | 3.2 | 3.4 |
| 2013 | 2.9 | 2.8 | 2.7 | 2.8 | 0.8 | 3.5 | 3.5 | 3.6 |
| 2014 | 3.2 | 3.1 | 3.1 | 3.2 | 0.8 | 3.9 | 3.8 | 3.9 |
| 2015 | 3.4 | 3.4 | 3.3 | 3.4 | 0.8 | 4.1 | 4.0 | 4.1 |
| 2016 | 3.6 | 3.5 | 3.5 | 3.6 | 0.8 | 4.3 | 4.2 | 4.3 |
| 2017 | 3.5 | 3.5 | 3.5 | 3.6 | 0.8 | 4.3 | 4.2 | 4.4 |
| 2018 | 3.7 | 3.7 | 3.6 | 3.8 | 0.8 | 4.4 | 4.2 | 4.5 |

**LCL – lower confidence limit, UCL - upper confidence limit *Age and sex adjusted prevalence divided by proportion diagnosed**
